# Episodic future thinking reduces temporal discounting in healthy adolescents

**DOI:** 10.1371/journal.pone.0188079

**Published:** 2017-11-22

**Authors:** Uli Bromberg, Maria Lobatcheva, Jan Peters

**Affiliations:** 1 Department of Systems Neuroscience, University Medical Center Hamburg-Eppendorf, Hamburg, Germany; 2 Department of Biological Psychology, University of Cologne, Cologne, Germany; Technion Israel Institute of Technology, ISRAEL

## Abstract

Episodic Future Thinking has proven efficient in reducing impulsive behavior in several adult populations. Whether it also has a beneficial impact on decision making in adolescents is not known. Here the impact of episodic future thinking on discounting behavior was investigated in a sample of healthy adolescents (n = 44, age range 13–16 years). Discounting behavior in trials including episodic future thinking was significantly less impulsive than in control trials (*t* = 2.74, *p* = .009, *d*_*z*_ = .44). In a subsample we controlled for executive function, alcohol use and developmental measures. Neither executive function nor alcohol use but developmental measures explained variability in the effect of episodic future thinking. These findings reveal that episodic future thinking can improve adolescent decision making while the effect is to some degree modulated by developmental measures.

## Introduction

Adolescents are often described as an especially impulsive population [1]. Their impulsive decisions can have both short- and long-term negative consequences, e.g. when experimenting with substances such as alcohol and nicotine: The majority of people who begin daily cigarette smoking during adolescence are addicted to nicotine well into adulthood [2], and the age of initiation of alcohol consumption predicts the degree of alcohol misuse in adult life [3]. Since adolescents may not take such future consequences into account when making decisions, it is important to investigate factors that can modulate adolescent impulsive decision making, thereby attenuating potentially harmful behavior.

Several previous studies both in adult and child populations have shown that Episodic Future Thinking can have a positive influence on impulsive decision making. In this study, we aimed at investigating this effect in an adolescent population.

One widely used measure of impulsivity is delay discounting behavior. In the delay discounting paradigm participants make a series of choices between smaller-but-sooner and larger-but-later rewards. People will generally prefer larger over smaller rewards, but imposing a delay typically induces a devaluation, thereby engaging participants to trade-off between value and time. Increased choices of smaller-sooner over larger-later rewards reflects increased impulsivity in this task. While discounting behavior is reasonably well characterized by a hyperbolic function [4], the degree of discounting varies considerably across individuals. In particular, populations suffering from addictive disorders, typically show increased discounting [5]. In line with their propensity for impulsive behavior [6], adolescents also show increased discounting compared to young adults [7]. And the trade-off between value and time invoked in the experimental discounting-paradigm resembles many situations in adolescent’s real life. E.g. indulging in immediately available social pleasures with peers, as opposed to studying hard in school, pursuing future educational goals. This measure is thus suitable for the characterization of impulse control in adolescence.

Episodic Future Thinking is one factor that has repeatedly proven successful in modifying delay discounting behavior. Episodic future thinking is the capacity to imagine personal future events, and is also known as mental time travel or prospection [8,9]. This capacity might be specific to humans, and is central for our ability to withstand short-term impulses in favor of long-term benefits [10,11]. Peters & Büchel (2010) [12] found that presenting short verbal cues referring to personal episodic future events (episodic tags) during a delay discounting task reduced discounting behavior. This effect (the episodic effect) was subsequently replicated a number of times, e.g. in healthy adults [13], in obese women and children [14,15], in alcohol dependent adults [16], and in patients with hippocampal amnesia [17]. A positive relation between the extent to which the episodic tags provoked mental associations, in the sense of evoking thoughts and / or feelings related to the event, and the size of the episodic effect [12], suggests that the effect to some degree relies on the ability and use of episodic future thinking. It has previously been shown that the ability to vividly imagine future events is related to delay discounting in adolescents [18]. However, to our knowledge no data has yet been reported on the episodic effect in adolescents. Because adolescents are both prone to show risk-seeking and impulsive behavior, and at the same time make a sensible target-group for prevention programs [19], it is particularly relevant to explore the episodic effect in the adolescent population. To this end, we investigated the episodic effect in a group of 44 adolescents aged 13 to 16. Supported by the previously observed association between episodic future thinking and delay discounting in adolescents [18], as well as previous successful use of episodic tags, not only in adults but also in a group of children [14], we expected to confirm the episodic effect in our adolescent sample. We also hypothesized the adolescent episodic effect to be related to the extent to which the episodic tags would invoke mental associations [12].

Besides investigating the episodic effect, we also investigated the influence of several control-variables on the effect, in a subsample of 32 adolescents. First, impulsive decision making is related to numerical age [7,20–22]. However, age-related changes in impulsive decision making during adolescence might be characterized more precisely by other measures than numerical age, such as physical maturation and gonadal hormonal levels [23,24]. These developmental measures are especially relevant during adolescence, including the age-range in our sample. We therefore extended the measure of numerical age with individual differences in self-reported physical development and circulating testosterone levels. Second, executive functions such as working memory have been shown to interact with the episodic effect [13,25]. To this end, we assessed individual differences in two measures of executive function. Finally, due to the robust association of problematic alcohol use with impulsive choice behavior [5], and with future thinking ability [26,27] we included individual differences in alcohol use in the control analysis.

Taken together, the aim of the present study was to investigate the effect of episodic future thinking on impulsive decision making in a sample of healthy adolescents. Furthermore, we controlled for the impact of several variables: development, executive function and alcohol use on the main effect in a control analysis. The aim of the study was achieved by showing the episodic effect in our sample of adolescents. Of the control-variables investigated, only developmental measures had an impact on the main effect.

## Materials and methods

### Participants

Data from 53 adolescents (age = 13–16 years, 21 male) was obtained. Participants, and their parents or legal guardians, provided informed written consent, and the study procedure was approved by the local ethics committee (Hamburg Board of Physicians). All participants were fluent German speakers and were predominantly from white families. Adolescents were recruited from three schools in a large City in northern Germany. Two researchers presented the study to eighth and ninth graders during class, and handed out recruitment packages. Students were encouraged to study the recruitment packages at home, together with their parents or legal guardians. Participants signed up via mail, E-mail or telephone. Exclusion criteria for adolescents included any diagnosed psychiatric disorder or estimated IQ < 70. Adolescent participants were also screened for physical health.

### General procedure

Data assessment was conducted individually in the same room, by one of two researchers, on two separate days. Each session lasted app. 1.5 to 2 hours, during the afternoon. On day one a pre-test delay discounting-task, an interview on personal episodic future events, saliva samples for testosterone-level and executive function constructs were assessed. On day two the episodic delay discounting-task, further saliva samples for testosterone-level, self-reported physical development and alcohol use were assessed. Participants were compensated for their participation with a fixed amount of € 40 and a variable amount between € 10 and € 15, depending on their choice behavior in the Episodic Discounting Task (see below).

### Pre-test delay discounting

On the first day of data assessment delay discounting was assessed using an adaptive computer-based procedure, previously used and described elsewhere [28]. Participants made choices between a smaller-sooner reward of 10 EUR available immediately and a larger-later reward of varying size, encompassing seven delays (1, 2, 7, 14, 30, 90 and 180 days). Using a staircase-procedure each participants’ indifference-points (ID-points) were determined. The staircase-procedure allows for a large range in larger-later reward sizes (range = 10,20–410,00 EUR, median = 18,50 EUR). These ID-points reflect the amount where the participant is expected to be indifferent to the SS and LL option. Individual discount rates were estimated as described below (Computational Modelling). The experiment was performed using the Presentation software package Version 17.1 (Neurobehavioral Systems Inc., Berkeley, CA). All rewards were hypothetical.

### Pre-test interview

On day one, participants were asked to report between five and 12 relevant personal events expected to take place soon. Specifically, events from the following time-windows were required: Within the following week, within the next 2–4 weeks, within the next 2–4 months, within the next 5–7 months. After documenting the events, participants were asked to rate each event for its personal relevance, valence and arousal on a six-point scale. Then, for each event, a short description was chosen (episodic tag), e.g. the event ‘In six weeks (42 days) I am going to visit my uncle’ could be tagged as ‘Visit_Uncle’. For the Episodic Discounting-task (see below) five to seven of these episodic tags were selected. Only tags with positive or neutral valence were included. Preference was given to tags high in relevance and arousal.

### Episodic discounting-task

**Fig 1:**
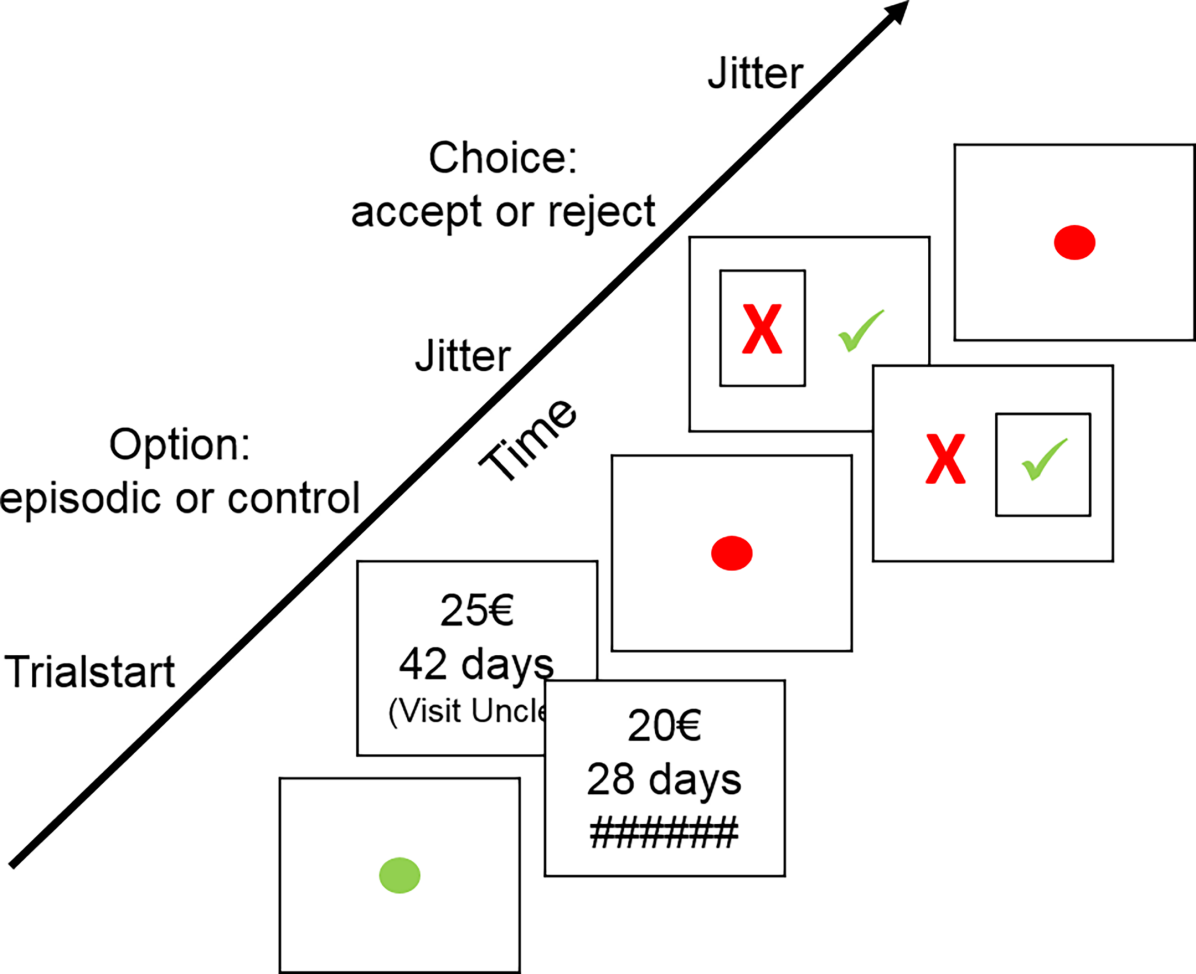
Episodic discounting-task. The Episodic Discounting task.

The individual discount rates that had been estimated based on the pre-test delay discounting-task were used to generate subject-specific trials in this episodic delay discounting-paradigm to test the episodic effect. Each participant completed 2 sessions containing 56 choices each (yielding a total of 112 trials), covering 7 delays, between 10 EUR available immediately and a larger reward available after a delay. For 3 participants, the 2 sessions contained only 54 choices each, covering 6 delays, because only 6 future events had been reported. The smaller-sooner reward, which was always the same, was known to the participants and was therefore not presented on the screen. The task contained two conditions: During half of the trials the larger amount available in euro (e.g. ‘25 EUR’) was presented together with the delay in days (e.g. ‘42 days’), as well as the timely matching episodic tag (e.g. ‘Visit_Uncle’). Importantly the delays presented would exactly match the reported timing of the episodic tag (episodic condition). In this sense the delays in this task differed somewhat between participants, but always stayed within the predefined time-windows, as described above on the Pre-test Interview. During the other half of the trials only the larger amount available was presented together with the delay (control condition), as well as a short line of the number sign (‘#####’), aiming at matching the perceptual visual stimuli of the episodic condition. The larger-later reward amounts were subject-specific to the individual discount-rates estimated based on the pretest discounting task, within a lower limit of € 10,50 and an upper limit of € 50,00 (Episodic condition: range = 10,50–49,60 EUR; Median = 21,40 EUR. Control condition: range = 10,50–49,70 EUR; Median = 20,20 EUR.) Importantly, the reward amount sizes do not differ between the two conditions ((*t*_(40)_ = 0.10, *p* = .90; CI = [-0.40 0.40]). The participants would make their choice by pressing the advised keys on a keyboard, and received visual feedback about their choice (Fig 1). The participants were instructed to perform the task in the same way as the pre-test delay discounting-task. Furthermore, they were informed that whenever a delay would match the previously reported events timewise, the episodic tag for that event would appear on the screen along with the larger-later reward, and that it would appear to help them discriminate the timing of the delay. No further instruction was given about the episodic tag, especially there was no explicit instruction to use future simulation. Participants were informed, that after finishing the task, the computer would randomly pick one choice of theirs per which they would be compensated in accordance with the choice made. Compensation was given as a gift card for Amazon and was either handed out immediately (if the SS had been chosen), or send via mail at the time of the chosen delay (if the LL had been chosen). The value of the gift card was limited to amounts between €10 and €15. After finishing the task participants rated for the episodic tags the degree to which it elicited episodic associations, and the vividness of these associations on a six-point scale. Individual discount-rates were estimated using the estimation procedures as described below (Computational Modelling).

### Developmental stage

In our subsample of 33 adolescents, developmental stage was characterized. First, we measured testosterone levels via saliva samples. To account for rhythmic fluctuations in circulating testosterone levels, three to four saliva samples were collected during each of the two assessment sessions. The saliva samples were pooled and analyzed by a local lab (Innovation Beyond Limits, Hamburg, Germany).

Second, we assessed self-reported physical development using the Pubertal Development Scale [29]. Both sexes reported growth in height, growth of body hair, changes in skin, and a self-evaluation whether development was considered early or late. Girls further reported their growth of breasts. Boys further reported changes in voice and changes in facial hair. Relative scores were calculated for each participant by dividing the individual score by maximum possible score (girls max. score = 21; boys max. score = 25, relative scores range from 0.2 to 1.0). Third, numerical age was assessed in days.

### Additional neuropsychological measures

In our subsample of 33 adolescents, we assessed Matrix Reasoning as well as Working Memory, using two subscales from the German version of the Wechsler Intelligence Scale for Children (WISC-IV) [30]. The subscale Matrix Reasoning assesses the capacity of fluid reasoning, requiring participants to fill in pictorial matrices of varying difficulty. Maximum score = 35. The subscale Digit Span assesses working memory, requiring participants to repeat dictated series of digits, forwards and backwards. Maximum score = 32.

### Substance use

In our subsample of 33 adolescents, we assessed the self-report questionnaire ESPAD (www.espad.org). This includes estimates of alcoholic units as well as alcohol-binges consumed in a life-time, during the last 12 months and during the last 30 days. Participants are asked to report the number of occasions where they have consumed alcohol. Alcohol-binges are defined as having 5 or more drinks on one occasion. The latest ESPAD-Report from 2015 (*www*.*espad*.*org*), covering data from 96046 students (age 15–16 years) from 35 European countries, report average consumption only in percentage as reported use independent of number of occasions, as follow: life-time alcohol use in 80% of the students (range across countries = 35–96%), a last 12 months use in 71% (range across countries = 24–90%), and a last 30 days use in 48% of students (range across countries = 9–73%). Alcohol-binges are reported for last 30 days in 13% of students (range across countries = 3–32). The number of occasions, which we use for our analysis, however are not reported. In our sample we see very similar use with life-time alcohol use in 75%; last 12 months alcohol use in 69%; last 30 days alcohol use in 41% and last 30 days alcohol binges in 9%. Descriptive statistics on the reported number of occasions of alcohol consumption in our sample is listed in Table 1.

**Table 1:** Descriptive statistics on reported alcohol consumption.

Number of occasions	Lifetime Alcohol	Last 12 months Alcohol	Last 30 days Alcohol	Lifetime Binge	Last 12 months Binge	Last 30 days Binge
0	8	2	2	11	0	0
1–2	7	11	11	4	5	5
3–5	9	5	5	3	1	1
6–9	2	1	1	1	3	3
10–19	2	1	1	0	1	1
20–39	0	3	3	3	1	1
>40	4	1	1	0	0	0

*Note*: Listed are the number of participants who reported the indicated ‘Number of Occasions’ in the alcohol- and binge-consumption categories.

### Sample and outliers

Of the 53 adolescent participants seven were excluded prior to data analysis due to technical faults in data assessment. One was dismissed due to nonsystematic indifference points in the pre-test delay discounting-task [31]. Furthermore, one outlier (i.e. scoring ± 3 SD from the *M*) was detected in discount-rate for the episodic condition. The main adolescent sample for analysis included data from 44 participants. In the subsample one outlier was detected in the episodic tag effect, leaving 32 participants for this additional analysis.

## Data analysis

Data analyses were conducted using R, version 3.2.3 (R Core Team, 2015) and the R-packages corrplot, version 0.73; ez, version 4.3; ggplot2, version 2.0.0; grid, version 3.2.3; gridextra, version 2.0.0; lmtest, version o.9-34; plotrix, version 3.6–1; plyr, version 1.8.3; psych, version 1.5.8 tidyr, version 0.4.0; Rstudio, version 0.98.1091 as well as Matlab (MathWorks, Natick, MA). Effect-sizes for paired samples are reported as Cohens *d*_*z*_, otherwise as Cohens *d* [32]. For the linear regression model, we used the *z*-transformed scores of variables involved.

### Computational modelling

For the discounting task data we approximated the devaluation of value over time using a hyperbolic model [33] in the form of:

$$\mathrm{SV}=\frac{R}{\left(1+k\;*\;D\right)}$$

where SV = subjective value; R = reward; D = delay in days; *k* = discount rate. We performed maximum likelihood parameter estimation [34] using optimization procedures implemented in Matlab (Mathworks, Natic, MA, fminsearch) with a softmax action selection function in the form of:

$$P_{\mathrm{chosen}}=\frac{\exp\left({\mathrm{SV}}_{\mathrm{chosen}}/temp\right)}{\exp\left({\mathrm{SV}}_{\mathrm{chosen}}/temp\right)+\exp\left({\mathrm{SV}}_{\mathrm{other}}/temp\right)}$$

where *P*chosen = probability of the chosen option; SVchosen = subjective value of the chosen option; SVother = subjective value of the other available option; *temp* = temperature, to fit the model to all trials from each participant. This estimation procedure provides estimates of each participant’s individual discount rate (*k*). The free parameter (*k*) is considered a direct behavioral measure of impulsivity, with higher values reflecting more impulsive choice behavior. The free parameter (*temp*), which describes the noise in the decision-making behavior, was not further analyzed.

In line with our hypothesis of attenuated delay discounting in the episodic condition, separate models were fit to the control and episodic trials.

**Fig 2:**
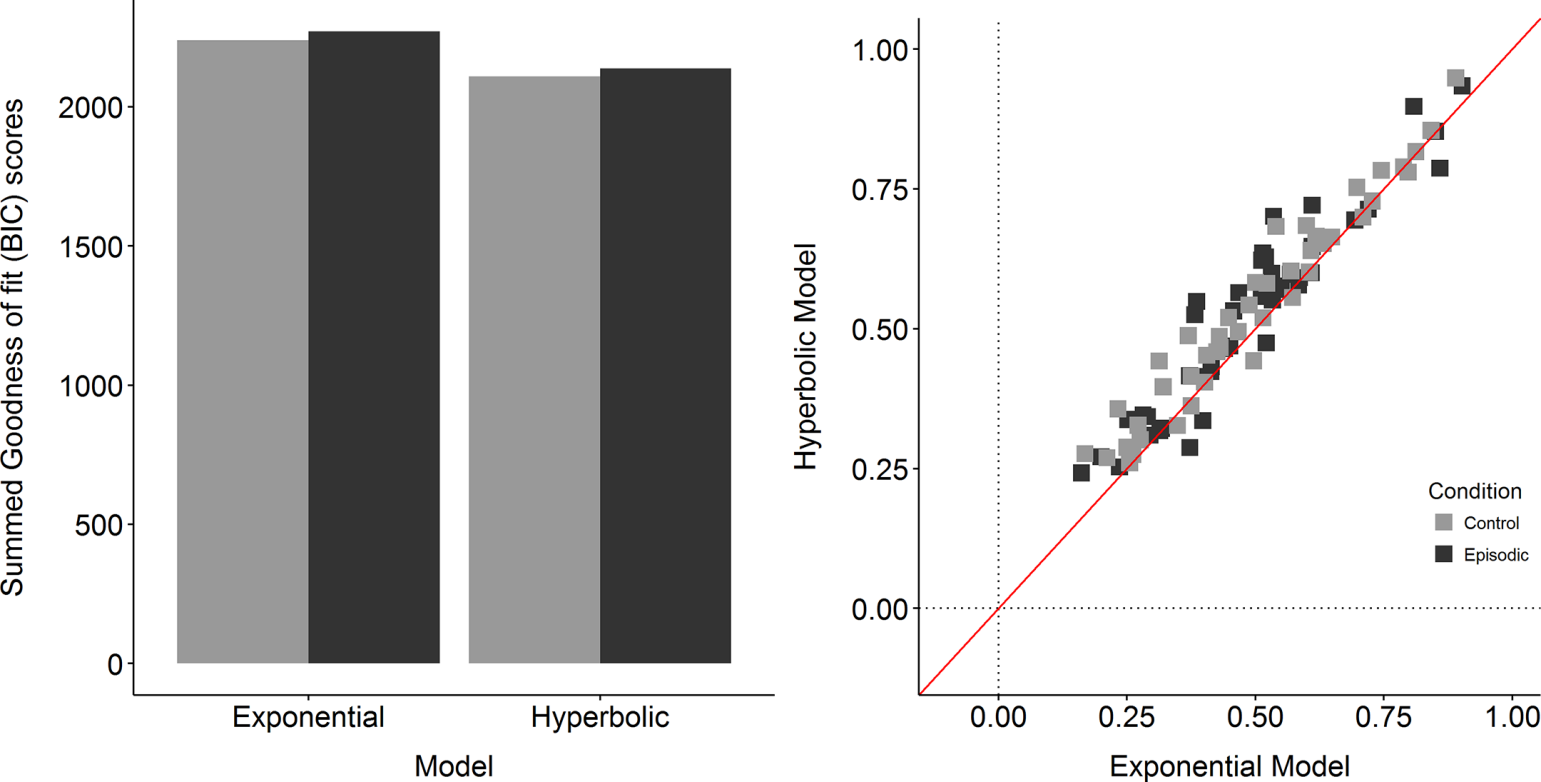
Goodness of fit for the exponential and the hyperbolic model. left: Summed BIC-scores as a Goodness of fit, comparing the exponential and the hyperbolic model by condition (light grey = control; dark grey = episodic); Fig 2 right: Individual Pseudo adjusted R2 values as a Goodness of fit, comparing the exponential (x-axis) and the hyperbolic (y-axis) model by condition (light grey = control; dark grey = episodic).

Furthermore, we compared the goodness of fit of the hyperbolic discounting model [33] to the fit of an exponential discounting model [35] in the form of:

$$\mathrm{SV}=A_{e}^{-bD}$$

for the Episodic Delay Discounting behavior with the Bayesian Information Criterion (BIC). The hyperbolic model overall proved a superior fit as compared to the exponential model (see Fig 2). For individual model fits we further calculated McFaddens adjusted Pseudo R2 in the form of

$$Pseudo\;adjR^{2}=1-\frac{\ln L_{1}-K}{\ln L_{0}}$$

Where *L*_*1*_ = the model of interest; *K* = number of model parameters; *L*_*0*_ = the zero model [36]. These proved good individual model fits with values > .25 and further proved the hyperbolic model fit to be superior to the exponential model fit (see Fig 2 right). Accordingly, after log-transformation, yielding appropriate distributions, the log-transformed estimated discount rates (*k*) from the hyperbolic model were used for further analyses.

### Model-free analysis

**Fig 3:**
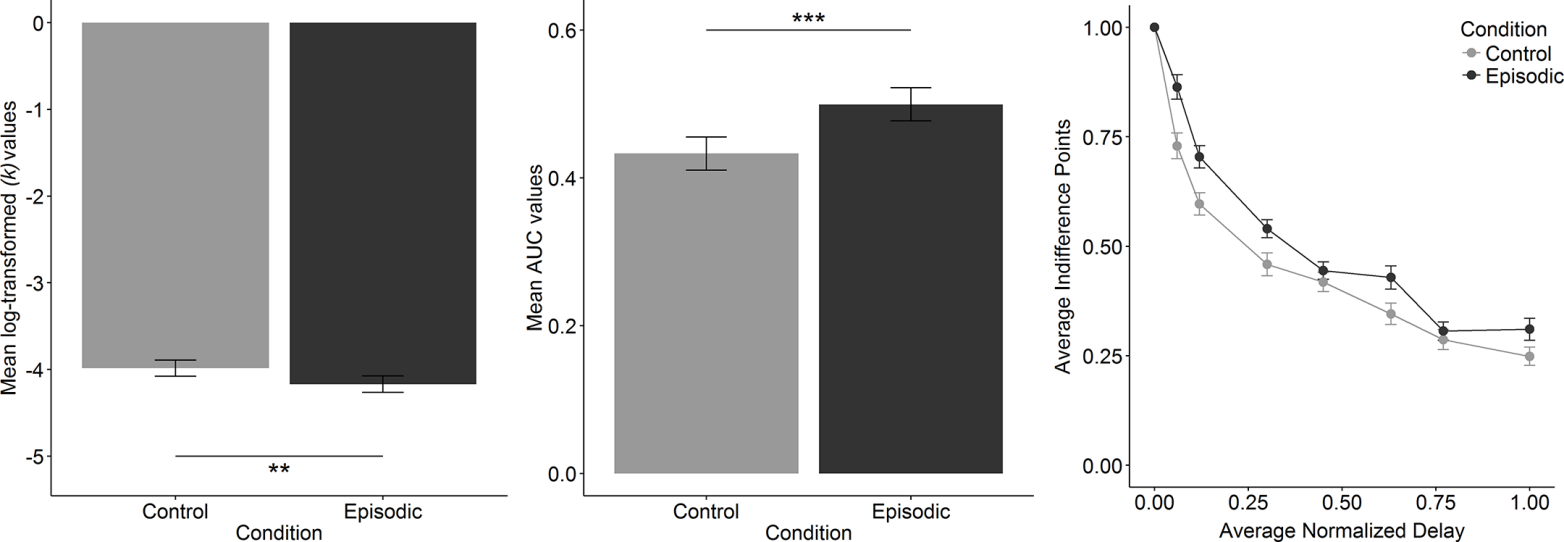
The episodic effect, n = 44. The episodic effect. Mean discount rate (parameter log(*k*) values); *p* = .009 (left); mean area under the curve (AUC) values; *p* < .001 (middle); n = 44. Averaged ID-points (right); n = 41.

To further test our main hypothesis with a model-free measure of impulsive discounting behavior, in line with the Area under the Curve approach (AUC) [37], we estimated ID-points of the Episodic Discounting Task behavior by fitting a logistic curve to the proportion of choices of the LL reward as a function of the LL amount, per delay and condition [38]. We then calculated the Area under the Curve for each participant per condition [37], which were used for testing our main hypothesis. All delays, which are specific to each participant based on their personal events, were normalized individually as a proportion of the longest delay per condition. For visualization of the AUC-Group effect (see Fig 3 (right)) we further averaged the ID-points for each normalized delay across all participants who had 7 delays. The 3 participants who only had 6 delays (see Episodic Discounting Task above) were not included in this graph. All 3 however confirm the overall relation AUC-episodic > AUC-control. Descriptive statistics are listed in Table 2.

**Table 2:** Descriptive statistics of discounting variables.

Sample	Measure	*M*	*SD*	Min	Max
Main Sample, n = 44	Episodic log(*k*)	-4.17	1.23	-6.75	-0.89
	Control log(*k*)	-3.98	1.26	-6.72	-0.57
	Episodic AUC	.50	.21	.09	.93
	Control AUC	.44	.22	.04	.93
Subsample, n = 32	Episodic log(*k*)	-4.04	1.16	-6.43	-1.47
	Control log(*k*)	-3.84	1.19	-6.72	-1.50
	Episodic effect	0.19	0.47	-1.03	1.53

*M* = mean; *SD* = standard deviation; Min = minimum range value; Max = maximum range value; Episodic log(*k*) = episodic condition, log-transformed parameter *k*; Control log(*k*) = control condition, log-transformed parameter *k*; AUC = Area under the curve; Episodic effect = episodic tag effect as the dependent variable for regression analysis.

## Results

The developmental measures were positively correlated: Age_testosterone-level, *r*_(32)_ = .39, 95% CI [.05, .65], *p* = .03; Age_physical developmental scale, *r*_(32)_ = .48, 95% CI [.15, .71], *p* = .01; physical developmental scale_testosterone level, *r*_(32)_ = .30, 95% CI [-.05, .59], *p* = .09. Sex differences in the developmental scores were found for testosterone-level, *Estimated Difference*_(32)_ = 1.13, 95% CI [.57, 1.69], *p* < .001. For the physical development scale a trend level difference was found, *Estimated Difference*_(30)_ = .60, 95% CI [-.07, 1.27], *p* = .08. (Reported scores are z-transformed). There was no sex difference in numerical age, measured in days. Due to the strong correlation between numerical age and the physical development scale, we aggregated these measures for further analyses, by averaging z-transformed raw scores into one measure of developmental stage [18]. No sex difference was found for developmental stage, the episodic effect, matrix reasoning or working memory.

The post-test ratings on vividness and occurrence of associations during the episodic condition were highly correlated, *r*_(42)_ = .78, 95% CI [.63, .87], *p* < .001. In line with Peters & Büchel (2010) [12] we aggregated these post-trial ratings across all events into a single imagery-score by averaging z-scored values. As the six subscales of the ESPAD were highly correlated, ranging from *r*_(26)_ = .64 to *r*_(26)_ = .95, all p < .001, we summarized overall substance use in a single mean score on alcohol use, across all sub-scores. There were no sex differences in imagery, nor in alcohol use. Descriptive statistics are listed in Table 3.

**Table 3:** Descriptive statistics of control variables for subsample (n = 32).

Variable	*M*	*SD*	Median	Min	Max
Matrix Reasoning	25.53	4.06	26.50	12.00	31.00
Working Memory	19.41	4.07	19.50	13.00	32.00
Numerical Age in days (years)	5413 (14.8)	261 (.72)	5432.5 (14.9)	4937 (13)	5842 (16)
Physical Development	.69	.14	.71	.40	.95
Testosterone Level	36.08	30.31	28.50	1.97	109.70
Alcohol Use (Aggregated)	.02	.92	-.24	-.95	2.30
Development (Aggregated)	.01	.87	.06	-1.83	1.50

*Note*: *M* = mean; *SD* = standard deviation; Min = minimum range value; Max = maximum range value; Aggregated measures (Alcohol Use and Development) are z-standardized.

### Main analysis

We tested our main hypothesis on the episodic effect (control condition discount-rate log(*k*) minus episodic condition discount-rate log(*k*)) with a two-tailed paired t-test. The episodic effect was significantly different from zero (*t*_(43)_ = 2.74, *p* = .009, *d*_*z*_ = .44, with a smaller log(*k*) in the episodic condition *M* = -4.17, *SD* = 1.23, as compared to the control condition *M* = -3.98, *SD* = 1.26). As a control of our result, we further tested the difference in AUC between the two conditions, also with a two-tailed paired t-test, which confirmed our result (*t*_(43)_ = 4.56, *p* = .00004, *d*_*z*_ = .65, with a larger AUC in the episodic condition *M* = 0.50, *SD* = 0.21, as compared to the control condition *M* = 0.44, *SD* = 0.22). A Bonferroni correction for multiple testing did not change the significance of the test results (*p* = .009 and *p* < .001, respectively) (Fig 3).

The association of compound imagery score and the episodic effect was positive but not significant (robust regression, *t*_(42)_ = 1.49, *p* = .14). To further investigate the imagery-scores we divided the sample with a median split into a high-imagery and a low-imagery group. The episodic effect in the high-imagery group was significantly larger than zero (one-sample t-test, *t*_(21)_ = 3.17, *p* = .01, *d* = .68), whereas it was not different from zero in the low-imagery group (one-sample t-test, *t*_(21)_ = .97, *p* = 1, *d* = .21). However, the difference between the two groups was not significant (Welch two-sample t-test, *t*_(40)_ = 1.25, *p* = .65, *d* = .38).

### Control analyses

To control for developmental stage, executive function and substance use, we first confirmed the main episodic effect in the sub-sample (n = 32) (one-tailed paired t-test: *t*_(31)_ = 2.28, *p* = .03, *d*_*z*_ = .40, replicating a lower log(*k*) in the episodic condition *M* = -4.04, *SD* = 1.16, as compared to the control condition *M* = -3.84, *SD* = 1.19).

We next set up a linear multiple regression model (with z-standardized variables) with the episodic effect as the dependent variable, and developmental stage, testosterone-level, sex, working memory, matrix reasoning and alcohol use as predictors. The episodic effect in the sub-sample was normally distributed, and there were no outliers in the sense of ± 3 *SD* from the mean. The model proved to significantly (*p* = .01) explain approximately 42% of the variability in the episodic effect (adjusted *R*^*2*^ = .42). Significant predictors were developmental stage, testosterone-level and the interaction-term sex*developmental stage. Sex showed a trend level effect (see Table 4). The sample size of sub-groups (dividing participants per developmental stage and sex) were too small (less than 5 participants per cell) to test the underlying nature of the interaction between developmental stage and sex. The predictors working memory, matrices and alcohol use were not significant.

**Table 4:** Regression analysis.

Predictor (z-scores)	Estimate	Standard Error	t-value	*p-value*
Model predicting the Episodic effect, (n = 32), adjusted *R*^*2*^ = .42				
Working memory	.008	.162	.05	.96
Matrix Reasoning	.044	.155	.28	.78
Alcohol use (Aggregated)	-.176	.155	-1.14	.27
Development (Aggregated)	1.361	.302	4.50	.00
Testosterone Level	-1.260	.313	-4.01	.00
Sex	-1.301	.647	-2.01	.06
Sex*Testosterone Level	.685	.802	.85	.40
Sex*Development	-1.710	.579	-2.96	.00
Development*Testosterone L.	-.210	.191	-1.07	.29
Sex*Dev.*Testosterone L.	-.718	.817	-.88	.39

Dev = Development. The symbol ‘*’ indicates an interactive effect between variables. All variables in the model were z-standardized.

## Discussion

We investigated the effect of episodic future thinking on delay discounting in healthy adolescents, by incorporating episodic future information in a delay discounting paradigm. We found that episodic tags (short verbal cues referring to real future events planned by the participant) reduced adolescent discounting behavior. Choice behavior was less impulsive in the episodic condition as compared to the control condition with an effect size of *d*_*z*_ = .44. An association between post-test imagery scores and the main effect was positive but not significant. Finally, in a subsample we show that the episodic effect is not significantly influenced by alcohol use or executive measures, but by developmental measures (pubertal development, numerical age and testosterone level), partly interacting with sex.

Most importantly we show that episodic tags attenuate adolescent delay discounting behavior. Trials that included a tag referring to a personal future event, were associated with reduced delay discounting compared to control trials without future event tags. The effect size of *d*_*z*_ = .44 found in our adolescent sample is similar to a previously published study in a group of young adults (*d* = .40) [12]. Our model-free AUC-control analysis confirms the effect, independently of the model used to describe the discounting behavior. While this and related episodic effects have been shown repeatedly in adult groups [13,15,39], the modification of adolescent delay discounting is of special interest. A recent study concluded that adolescent impulsivity is determined by the adolescent’s relative future orientation, rather than their sensitivity to immediate rewards [40]. In another study individual differences in future time perspective as measured by self-report were negatively associated with delay discounting behavior in a large sample of 14 to 22 year old participants [41]. Also, adolescents who are high in general episodic future thinking ability discount future rewards less steeply [18]. These studies emphasize the value of enhancing adolescent future orientation in decision making, which is further supported by our finding of an episodic effect in adolescents. Episodic future thinking has previously proven successful in increasing self-control in individuals with self-control deficits such as eating disorders [15]. Similarly, Bickel et al. (2015) [42] suggested to add episodic future thinking training in addiction treatment, aiming at increasing self-control. This approach proved effective in a group of 50 alcohol dependent adults [16], where future related cues induced less impulsive behavior, also related to the substance of abuse. Following this line of thought, our results suggest that further investigation of episodic future thinking as a potential tool in substance abuse prevention in adolescence might be warranted [19].

In our adolescent sample we did not replicate the previously reported association between the episodic effect and post-trial imagery-scores [12]. The estimated regression parameter was positive, and a median-split analysis showed that the episodic effect of those high in imagery differed significantly from zero, whereas the episodic effect of those low in imagery did not differ significantly from zero. While our data does point towards the same direction as in Peters & Büchel, (2010) [12], this association might be too small to be detected in our entire sample. Future investigations into the episodic effect may want to add further controls, besides the ratings used here, to clarify what quality of the future episodic events trigger the effect.

Our regression-model, including the covariates developmental stage, executive function, testosterone level, sex and alcohol use, proved an overall significant influence on the episodic effect (Table 4). The positive influence by developmental stage is dependent on the interaction with sex. However, our sample-size is too small to evaluate any sub-samples (divided by sex and developmental stage), and draw any conclusions about the nature of this interaction, which calls for further studies with larger sized subgroups. The effect of testosterone-level on the episodic effect was negative. The role of endocrine parameters in adolescent decision making is presently not well understood [43], and the literature on the relation between testosterone-level and discounting behavior is sparse. While one animal study reported decreased discounting in testosterone-treated rats compared to control rats [44], adolescent humans high in testosterone level contrarily showed a trend towards increased discounting [18]. Studies in healthy young male students showed that cutaneous administration of topical testosterone gel did not influence discounting behavior [45], whereas higher testosterone levels in non-impulsive subjects did correlate with increased discounting behavior [46]. Whether increased levels of testosterone may boost impulsive decision making, thereby potentially attenuating the effect of episodic tags can only be speculated, and would need to be evaluated in further studies. Contrarily to previous results in adults where the episodic effect was stronger for participants high in working memory [13], executive function did not modulate the episodic effect in our adolescent sample, possibly due to these functions not being fully developed until young adulthood [47]. Also the tasks used here might tap into different aspects of executive function than the task used by Lin & Epstein (2014) [13]. While alcohol abuse is positively related to discounting behavior [5] and EFT ability has been shown to be negatively related to problematic alcohol use [26,27] our data show no influence of alcohol use on the episodic tag effect. This contrasts to a report by Snider et al., [16] where a beneficial impact of EFT on discounting behavior was more readily apparent in alcohol dependent individuals with less severe abuse, showing some modulation of the episodic effect by alcohol use. Our sample of adolescents however differ strongly from the alcohol dependent sample in Snider et al., being much younger and without individual substance abuse diagnostics. Furthermore, the self-reported use of alcohol in our sample is relatively low (with approximately half of our participants in the lower quarter of the distribution, see Tables 1 and 2), and is likely too low to detect a potential relation with the episodic effect.

Some limitations in our study need to be acknowledged. The use of number-signs in our control condition can only to some degree match the perceptual qualities of the visual experimental stimulus (the personal events). In line with Peters & Büchel (2012) [12], qualitative characteristics of the experimental condition, especially the saliency of these personal events, were not matched using this relatively simple control condition. Other studies that also show the episodic effect have used more elaborate control conditions, such as engagement in events from non-personal stories [15,39], or personal recent or present events [13,14], attempting to match the qualitative features of the experimental stimuli. These studies confirm the episodic effect even when using such elaborate control conditions. Future studies on the episodic effect in adolescents might benefit from the inclusion of such additional control conditions. The gold-standard for assessing physical development, namely conducting an examination by a physician [48], was beyond the scope of our study. Finally, our exploratory regression analysis, controlling for several potential modulators, proved that our sample size was too small to draw any conclusions about the interaction between developmental stage and sex.

In summary, we show an episodic effect in adolescents, similar in size to the effect previously reported in healthy young adults [12]. This finding supports the potential of further investigating episodic future thinking as a factor that can support improving health-related choice behavior [15,42]. Crucially, showing this effect in an adolescent sample extends the potential of episodic future thinking to be useful also in preventive work with young subjects, before addictive disorders actually develop [19,49].

## Supporting information

S1 Dataset Individual parameters. (TXT)

S2 Dataset Individual parameters (subsample). (TXT)

S3 Dataset Normed delays. (TXT)

## References

[1] Steinberg L. A social neuroscience perspective on adolescent risk-taking. Dev Rev. 2008;28: 78–106. doi: 10.1016/j.dr.2007.08.002 18509515 10.1016/j.dr.2007.08.002 PMC2396566

[2] U.S. Department of Health and Human Services. Health, United States: With Special Feature on Emergency Care. Hyattsville, MD.; 2013.

[3] Dawson DA, Goldstein RB, Patricia Chou S, June Ruan W, Grant BF. Age at First Drink and the First Incidence of Adult-Onset DSM-IV Alcohol Use Disorders. Alcohol Clin Exp Res. 2008;32: 2149–2160. doi: 10.1111/j.1530-0277.2008.00806.x 18828796 10.1111/j.1530-0277.2008.00806.x PMC2760820

[4] Green L, Myerson J, Macaux EW. Temporal Discounting When the Choice Is Between Two Delayed Rewards. J Exp Psychol Learn Mem Cogn. 2005;31: 1121–1133. doi: 10.1037/0278-7393.31.5.1121 16248754 10.1037/0278-7393.31.5.1121

[5] MacKillop J, Amlung MT, Few LR, Ray LA, Sweet LH, Munafò MR. Delayed reward discounting and addictive behavior: a meta-analysis. Psychopharmacology (Berl). 2011;216: 305–321. doi: 10.1007/s00213-011-2229-0 21373791 10.1007/s00213-011-2229-0 PMC3201846

[6] Kim-Spoon J, Kahn R, Deater-Deckard K, Chiu PH, Steinberg L, King-Casas B. Risky decision making in a laboratory driving task is associated with health risk behaviors during late adolescence but not adulthood. Int J Behav Dev. 2016;40: 58–63. doi: 10.1177/0165025415577825 26770006 10.1177/0165025415577825 PMC4707653

[7] Olson EA, Hooper CJ, Collins P, Luciana M. Adolescents’ performance on delay and probability discounting tasks: Contributions of age, intelligence, executive functioning, and self-reported externalizing behavior. Personal Individ Differ. 2007;43: 1886–1897. doi: 10.1016/j.paid.2007.06.016 18978926 10.1016/j.paid.2007.06.016 PMC2083651

[8] Addis DR, Wong AT, Schacter DL. Remembering the past and imagining the future: Common and distinct neural substrates during event construction and elaboration. Neuropsychologia. 2007;45: 1363–1377. doi: 10.1016/j.neuropsychologia.2006.10.016 17126370 10.1016/j.neuropsychologia.2006.10.016 PMC1894691

[9] Gilbert DT, Wilson TD. Prospection: Experiencing the Future. Science. 2007;317: 1351–1354. doi: 10.1126/science.1144161 17823345 10.1126/science.1144161

[10] Bar M. The proactive brain: memory for predictions. Philos Trans R Soc B Biol Sci. 2009;364: 1235–1243. doi: 10.1098/rstb.2008.0310 19528004 10.1098/rstb.2008.0310 PMC2666710

[11] Suddendorf T, Addis DR, Corballis MC. Mental time travel and the shaping of the human mind. Philos Trans R Soc B Biol Sci. 2009;364: 1317–1324. doi: 10.1098/rstb.2008.0301 19528013 10.1098/rstb.2008.0301 PMC2666704

[12] Peters J, Büchel C. Episodic Future Thinking Reduces Reward Delay Discounting through an Enhancement of Prefrontal-Mediotemporal Interactions. Neuron. 2010;66: 138–148. doi: 10.1016/j.neuron.2010.03.026 20399735 10.1016/j.neuron.2010.03.026

[13] Lin H, Epstein LH. Living in the moment: Effects of time perspective and emotional valence of episodic thinking on delay discounting. Behav Neurosci. 2014;128: 12–19. doi: 10.1037/a0035705 24512061 10.1037/a0035705 PMC4049454

[14] Daniel TO, Said M, Stanton CM, Epstein LH. Episodic future thinking reduces delay discounting and energy intake in children. Eat Behav. 2015;18: 20–24. doi: 10.1016/j.eatbeh.2015.03.006 25863227 10.1016/j.eatbeh.2015.03.006 PMC6504176

[15] Daniel TO, Stanton CM, Epstein LH. The Future Is Now Reducing Impulsivity and Energy Intake Using Episodic Future Thinking. Psychol Sci. 2013;24: 2339–2342. doi: 10.1177/0956797613488780 24022653 10.1177/0956797613488780 PMC4049444

[16] Snider SE, LaConte SM, Bickel WK. Episodic Future Thinking: Expansion of the Temporal Window in Individuals with Alcohol Dependence. Alcohol Clin Exp Res. 2016;40: 1558–1566. doi: 10.1111/acer.13112 27246691 10.1111/acer.13112 PMC5497459

[17] Kwan D, Craver CF, Green L, Myerson J, Gao F, Black SE, et al Cueing the personal future to reduce discounting in intertemporal choice: Is episodic prospection necessary? Hippocampus. 2015;25: 432–443. doi: 10.1002/hipo.22431 25676022 10.1002/hipo.22431

[18] Bromberg U, Wiehler A, Peters J. Episodic Future Thinking Is Related to Impulsive Decision Making in Healthy Adolescents. Child Dev. 2015;86: 1458–1468. doi: 10.1111/cdev.12390 26110500 10.1111/cdev.12390

[19] Gray JC, MacKillop J. Impulsive delayed reward discounting as a genetically-influenced target for drug abuse prevention: a critical evaluation. Psychol Clin Settings. 2015; 1104 doi: 10.3389/fpsyg.2015.01104 10.3389/fpsyg.2015.01104 PMC4554956 26388788

[20] de Water E, Cillessen AHN, Scheres A. Distinct Age-Related Differences in Temporal Discounting and Risk Taking in Adolescents and Young Adults. Child Dev. 2014;85: 1881–1897. doi: 10.1111/cdev.12245 24749521 10.1111/cdev.12245

[21] Scheres A, Dijkstra M, Ainslie E, Balkan J, Reynolds B, Sonuga-Barke E, et al Temporal and probabilistic discounting of rewards in children and adolescents: Effects of age and ADHD symptoms. Neuropsychologia. 2006;44: 2092–2103. doi: 10.1016/j.neuropsychologia.2005.10.012 16303152 10.1016/j.neuropsychologia.2005.10.012

[22] Steinberg L, Graham S, O’Brien L, Woolard J, Cauffman E, Banich M. Age Differences in Future Orientation and Delay Discounting. Child Dev. 2009;80: 28–44. doi: 10.1111/j.1467-8624.2008.01244.x 19236391 10.1111/j.1467-8624.2008.01244.x

[23] Dorn LD, Biro FM. Puberty and Its Measurement: A Decade in Review. J Res Adolesc. 2011;21: 180–195. doi: 10.1111/j.1532-7795.2010.00722.x

[24] Forbes EE, Dahl RE. Pubertal development and behavior: Hormonal activation of social and motivational tendencies. Brain Cogn. 2010;72: 66–72. doi: 10.1016/j.bandc.2009.10.007 19942334 10.1016/j.bandc.2009.10.007 PMC3955709

[25] Ward AM. A Critical Evaluation of the Validity of Episodic Future Thinking: A Clinical Neuropsychology Perspective. Neuropsychology. 2016; doi: 10.1037/neu0000274 26901169 10.1037/neu0000274

[26] Heffernan TM. The impact of excessive alcohol use on prospective memory: a brief review. Curr Drug Abuse Rev. 2008;1: 36–41. 19630703 10.2174/1874473710801010036

[27] Griffiths A, Hill R, Morgan C, Rendell PG, Karimi K, Wanagaratne S, et al Prospective memory and future event simulation in individuals with alcohol dependence. Addict Abingdon Engl. 2012;107: 1809–1816. doi: 10.1111/j.1360-0443.2012.03941.x 22578026 10.1111/j.1360-0443.2012.03941.x

[28] Peters J, Büchel C. Overlapping and Distinct Neural Systems Code for Subjective Value During Intertemporal and Risky Decision Making. J Neurosci. 2009;29: 15727–15734. doi: 10.1523/JNEUROSCI.3489-09.2009 20016088 10.1523/JNEUROSCI.3489-09.2009 PMC6666169

[29] Petersen AC, Crockett L, Richards M, Boxer A. A self-report measure of pubertal status: Reliability, validity, and initial norms. J Youth Adolesc. 1988;17: 117–133. doi: 10.1007/BF01537962 24277579 10.1007/BF01537962

[30] Daseking M, Petermann U, Petermann F. Intelligenzdiagnostik mit dem HAWIK-IV. Kindh Entwickl. 2007;16: 250–259. doi: 10.1026/0942-5403.16.4.250

[31] Johnson MW, Bickel WK. An Algorithm for Identifying Nonsystematic Delay-Discounting Data. Exp Clin Psychopharmacol. 2008;16: 264–274. doi: 10.1037/1064-1297.16.3.264 18540786 10.1037/1064-1297.16.3.264 PMC2765051

[32] Lakens D. Calculating and reporting effect sizes to facilitate cumulative science: a practical primer for t-tests and ANOVAs. Front Psychol. 2013;4 doi: 10.3389/fpsyg.2013.00863 24324449 10.3389/fpsyg.2013.00863 PMC3840331

[33] Mazur JE. An adjusting procedure for studying delayed reinforcement Quantitative analyses of behavior. Hillsdale, NJ: Erlbaum; 1987 pp. 55–73.

[34] Peters J, Miedl SF, Büchel C. Formal Comparison of Dual-Parameter Temporal Discounting Models in Controls and Pathological Gamblers. PLoS ONE. 2012;7: e47225 doi: 10.1371/journal.pone.0047225 23226198 10.1371/journal.pone.0047225 PMC3511467

[35] Samuelson PA. A Note on Measurement of Utility. Rev Econ Stud. 1937;4: 155–161. doi: 10.2307/2967612

[36] McFadden D. Conditional logit analysis of qualitative choice behavior In: Zarembka P. (ed) Frontiers in Econometrics. Academic Press; 1974 pp. 105–142

[37] Myerson J, Green L, Warusawitharana M. Area under the curve as a measure of discounting. J Exp Anal Behav. 2001;76: 235–243. doi: 10.1901/jeab.2001.76-235 11599641 10.1901/jeab.2001.76-235 PMC1284836

[38] Kable JW, Glimcher PW. An “As Soon As Possible” Effect in Human Intertemporal Decision Making: Behavioral Evidence and Neural Mechanisms. J Neurophysiol. 2010;103: 2513–2531. doi: 10.1152/jn.00177.2009 20181737 10.1152/jn.00177.2009 PMC2867580

[39] Daniel TO, Stanton CM, Epstein LH. The future is now: Comparing the effect of episodic future thinking on impulsivity in lean and obese individuals. Appetite. 2013;71: 120–125. doi: 10.1016/j.appet.2013.07.010 23917063 10.1016/j.appet.2013.07.010 PMC4185182

[40] Bos W van den, Rodriguez CA, Schweitzer JB, McClure SM. Adolescent impatience decreases with increased frontostriatal connectivity. Proc Natl Acad Sci. 2015;112: E3765–E3774. doi: 10.1073/pnas.1423095112 26100897 10.1073/pnas.1423095112 PMC4517266

[41] Romer D. Adolescent risk taking, impulsivity, and brain development: Implications for prevention. Dev Psychobiol. 2010;52: 263–276. doi: 10.1002/dev.20442 20175097 10.1002/dev.20442 PMC3445337

[42] Bickel WK, Quisenberry AJ, Moody L, Wilson AG. Therapeutic Opportunities for Self-Control Repair in Addiction and Related Disorders Change and the Limits of Change in Trans-Disease Processes. Clin Psychol Sci. 2015;3: 140–153. doi: 10.1177/2167702614541260 25664226 10.1177/2167702614541260 PMC4314724

[43] Crone EA, Dahl RE. Understanding adolescence as a period of social–affective engagement and goal flexibility. Nat Rev Neurosci. 2012;13: 636–650. doi: 10.1038/nrn3313 22903221 10.1038/nrn3313

[44] Wood RI, Armstrong A, Fridkin V, Shah V, Najafi A, Jakowec M. ‘Roid rage in rats? Testosterone effects on aggressive motivation, impulsivity and tyrosine hydroxylase. Physiol Behav. 2013;0: 6–12. doi: 10.1016/j.physbeh.2012.12.005 10.1016/j.physbeh.2012.12.005 PMC3615053 23266798

[45] Ortner GR, Wibral M, Becker A, Dohmen T, Klingmüller D, Falk A, et al No evidence for an effect of testosterone administration on delay discounting in male university students. Psychoneuroendocrinology. 2013;38: 1814–1818. doi: 10.1016/j.psyneuen.2012.12.014 23339890 10.1016/j.psyneuen.2012.12.014

[46] Takahashi T, Sakaguchi K, Oki M, Homma S, Hasegawa T. Testosterone levels and discounting delayed monetary gains and losses in male humans. Neuro Endocrinol Lett. 2006;27: 439–444. 16891992

[47] Sander MC, Lindenberger U, Werkle-Bergner M. Lifespan age differences in working memory: a two-component framework. Neurosci Biobehav Rev. 2012;36: 2007–2033. doi: 10.1016/j.neubiorev.2012.06.004 22771333 10.1016/j.neubiorev.2012.06.004

[48] Shirtcliff EA, Dahl RE, Pollak SD. Pubertal Development: Correspondence Between Hormonal and Physical Development. Child Dev. 2009;80: 327–337. doi: 10.1111/j.1467-8624.2009.01263.x 19466995 10.1111/j.1467-8624.2009.01263.x PMC2727719

[49] Story GW, Vlaev I, Seymour B, Darzi A, Dolan RJ. Does temporal discounting explain unhealthy behavior? A systematic review and reinforcement learning perspective. Front Behav Neurosci. 2014;8: 76 doi: 10.3389/fnbeh.2014.00076 24659960 10.3389/fnbeh.2014.00076 PMC3950931

